# Editorial: Pluripotent stem cell engineered 3D structures for disease modeling and tissue repairing

**DOI:** 10.3389/fncel.2023.1146143

**Published:** 2023-02-03

**Authors:** Liang Qiang, Michael A. Lane, Claudia A. Doege, Orly Reiner, Itzhak Fischer

**Affiliations:** ^1^Department of Neurobiology and Anatomy, Drexel University College of Medicine, Philadelphia, PA, United States; ^2^Marion Murray Spinal Cord Injury Research Center (MMSCRC), Drexel University College of Medicine, Philadelphia, PA, United States; ^3^Department of Pathology and Cell Biology, Columbia University, New York, NY, United States; ^4^Department of Molecular Genetics and Molecular Neuroscience, Weizmann Institute of Science, Rehovot, Israel

**Keywords:** 3D structure, organoid, hiPSC, cellular reprogramming, disease modeling, cell therapy

Three-dimensional (3D) culture of bioengineered tissues can be referred to as micro-tissues (3D aggregation of cells, *via* cell-cell contact or cell-extracellular matrix interaction), “organoids” (resembling an organ, typically “developed” following the spatial and structural cues), or assembloids (fusing of regionally distinct organoids, such as brain and spinal cord, or spinal cord and muscle). Engineered 3D tissues with central nervous system (CNS) identity are often developed from human pluripotent stem cells (hPSCs). Based on a variety of established culturing protocols, these 3D structures can be generated with an orderly cellular organization that not only contains essential cell components implicated in CNS development but incorporates complex cell-cell interactions. Indeed, they provide a novel platform for modeling CNS diseases and therapeutic cell replacement. With support of the revolutionary cellular reprogramming technology, complex 3D cellular structures derived from patients made a great leap forward in realizing personalized medicine for neurological disorders and injuries. This Research Topic aimed to collect and highlight recent advances and discoveries in the research arena of using hPSC-derived 3D cultures composed of various CNS cellular elements for disease modeling and cell therapy. This special issue includes six articles, three reviews, and three original research articles from excellent researchers in the field which covers some of the most recent and exciting findings in advancement of the human 3D CNS tissue engineering, as well as its applications in translational medicine.

The review article by O'Hara-Wright et al. entitled “Bioelectric Potential in Next-Generation Organoids: Electrical Stimulation to Enhance 3D Structures of the Central Nervous System” describes a cutting-edge technology to eradicate the present shortcomings of the generation of CNS organoids. Application of bioelectricity and electrical stimulation for the generation of a more versatile model of CNS organoids is proposed. The authors start off the review by paying homage to the updated biochemical (growth factors) and biomechanical (Matrigel) changes made to the PSC protocols and how it has revolutionized the field. They also strengthen their stance on introducing bioelectricity as the next revolutionary change to have a better physiologically accurate organoid. A thorough literature review is made on the potential of bioelectricity and modulation of endogenous electric field on development and differentiation, cell viability and proliferation, and other molecular mechanisms. Nevertheless, this review also highlights that the lack of bioelectric components in the current protocols is due to the knowledge gap in mapping the electric network of the body and the strength and duration of electric field used for stimulation. In all, the authors elucidate the advantage of using bioelectricity to generate physiologically relevant CNS organoids for developmental studies and disease modeling.

The minireview article by Iyer and Ashton entitled “Bioengineering the human spinal cord” aims to elucidate the nature of stem cell derived spinal organoids in the context of spinal cord neurodevelopment, particularly as it pertains to patterning along the rostrocaudal and dorsoventral axes. Rostrocaudal identity, which is mainly characterized by *HOX* profiles, can be tuned by manipulating organoid exposure to and timing of SMAD inhibitors with shorter exposures leading to more caudal identities, and can be further enhanced through patterning with FGF, WNT, GDF11, and RA. Dorsoventral patterning on the other hand relies on local concentration-dependent SHH and BMP signaling, which promotes the emergence of region specific progenitors that will then mature into organized neuronal domains. Limitations of generating human spinal organoids are also discussed as lack of standardized hiPSC culturing methods which results in limited cell diversity and organization, inconsistent reproducibility, and restricted culture durations. Current bioengineering strategies are proposed to leverage biomaterial-supported constructs, microfluidics gradients, and genetic engineering to overcome some of these limitations by providing a more dynamic range of environmental cues, including mechanoregulation, complex morphogenetic gradients, and spatially localized optogenetic signaling. Nevertheless, the authors summarize the standardization of culture techniques and further innovation of bioengineering strategies will continue to develop human spinal cord organoids as a useful system for studying patient-specific neurodegenerative diseases and will enable finding appropriate translational solutions.

In “Diseased, differentiated and difficult: Strategies for improved engineering of *in-vitro* neurological systems” Elder et al. review cellular reprogramming strategies for disease modeling and cell therapies. The authors highlight two distinct strategies to generate neurons from human PSCs–directed and transcription factor induced differentiation. Directed differentiation mimics the developmental process of neurogenesis by employing exogenous stimulations, whereas transcription factor induction utilizes genetic and epigenetic engineering tools to manipulate the activation and inactivation of master regulators in neurogenesis for various neuronal phenotypes. Some major limitations discussed by the authors include differentiating region-specific cell types and difficulty in maintaining their functionality over time to accurately model neurological diseases. However, with the expansion in cellular engineering, stem cell technology, and our understanding of the human brain, more hybrid approaches that combine aspects of directed and induced differentiation, co-culture, and organoids can overcome some of the limitations and prove beneficial in a technical and clinical setting.

The original research article by Miranda et al. entitled “A Dynamic 3D Aggregate-Based System for the Successful Expansion and Neural Induction of Human Pluripotent Stem Cells” depicts novel methods of expanding phenotypically stable hiPSCs in 3D cultures and effective differentiation of neural progenitors with specific region identities on a large scale. The authors discovered that with the help of orbital shakers or vertical-wheel bioreactors, hiPSC aggregates cultured in Gibco StemScale PSC medium exponentially expanded within a few passages. Furthermore, these hiPSC aggregates can be robustly differentiated into neural progenitor cells when cultured in either Neural Induction Medium (NIM) or N2B27 medium supplemented with dual SMA inhibition, but with somewhat different outcomes. For example, N2B27 aggregates display a larger size; and NIM yielded similar proportions of the three cell types (neurons, astrocytes, and immature neurons), whereas N2B27 promoted mostly neurons and immature neurons along with much fewer astrocytes. Interestingly, the single-cell calcium imaging which is to evaluate the functionality of the cells shows cells cultured in NIM were more responsive than cells cultured in N2B27. Lastly, the authors validated their protocols of generating neural progenitor cells with region identities. In conclusion, these methods are important for scaling up the hiPSC and their neuronal derivatives which potentially can be used in the clinical that depends on the scalability such as cellular therapies and drug screening.

Yates et al. report novel results from experiments utilizing hiPSCs derived from Veterans with and without Gulf War Illness (GWI) to establish cerebral organoids for disease modeling. In “Veteran-derived cerebral organoids display multifaceted pathological defects in studies on Gulf War Illness”, characterizing organoids after exposure to Gulf War toxicants revealed increased astrocytic reactivity, enhanced phosphorylation of tau proteins, decreased microtubule stability, and impaired neurogenesis were identified. Interestingly, some of these phenotypes were more pronounced in the organoids derived from the GWI Veterans, potentially indicating a susceptibility in these patients. In all, these results suggest that GW Veteran-derived human cerebral organoids not only can be used as an innovative human model to uncover the cellular responses to GW toxicants but can also serve as a platform for developing personalized medicine approaches for the veterans.

The original research article by Romero et al. entitled “Oligodendrogenesis and myelination tracing in CRISPR/Cas9-engineered brain organoids” puts forward an efficient approach to study the processes of oligodendrogenesis and myelination using CRISPR/Cas9 gene editing to knock in a fluorescent protein at the stop codon of PLP1, which is a marker for oligodendrocytes, thus creating a fusion protein to track the differentiation, migration, and maturation of OL cells in cerebral organoids. The authors demonstrated the efficacy of reverse knock-in when compared to forward knock-in when transfecting cells and performed quality control steps such as off-target screening and chromosome aberration assays to ensure high knock-in cell fidelity and minimal genetic aberration. This methodology in this article has the potential to be used for high-fidelity insertion of fluorescent tags into hiPSCs to study the development of certain cell types. The authors also validated the applicability of their model to effective drug screening by using cuprizone (CPZ), which is known to cause demyelination *in vivo*.

Overall, this Research Topic summarizes and highlights important findings related to the studies using human induced pluripotent stem cells for translational medicine and provides prospective advances in its application. Bioengineered human 3D cellular tissues have great potential in meeting growing need for more advanced disease modeling platforms and therapeutic cellular resources. Organoids, assembloids, circuitoids, and organ-on-a-chip systems are several modeling strategies that allow for great flexibility and ingenuity in mechanistic studying for organ development, as well as fostering personalized medicine disease treatment.

## Author contributions

LQ wrote this editorial. MAL, CAD, OR, and IF edited this editorial. All authors have approved it for publication.

